# Marine-derived magnetic nanocatalyst in sustainable ultrasound-assisted synthesis of 2,3-diphenyl-2,3-Dihydroquinazolin-4(1H)-One derivatives

**DOI:** 10.1016/j.heliyon.2024.e38948

**Published:** 2024-10-05

**Authors:** Foad Buazar, Mohammad Hosein Sayahi

**Affiliations:** a Department of Marine Chemistry, Khorramshahr University of Marine Science and Technology, P.O. Box 669, Khorramshahr, Iran; b Department of Chemistry, Payame Noor University, Tehran, Iran

**Keywords:** Biosynthesis, Catalyst, Magnetite nanoparticles, Organic synthesis, Ultrasound

## Abstract

This research presents a sustainable approach to fabricate iron oxide nanoparticles by employing phytochemicals derived from marine grass extract as both reducing and stabilizing agents. Formation of magnetic nanoparticles (MNPs) initially confirmed by ultraviolet–visible spectroscopy (UV–vis) showing absorption peak at 370 nm. X-ray diffraction (XRD) and transmission electron microscopy (TEM) techniques unveiled magnetic iron oxide NPs with a rod shape, an average size of 21 nm, and an inverse spinel crystal structure. The participation of organic compounds in the production and stabilization of MNPs was evidenced through Fourier transform infrared (FTIR) spectroscopy and thermogravimetric analysis (TGA). A vibrating sample magnetometer (VSM) demonstrated the magnetic properties of iron oxide NPs showing a saturation magnetization value of 21.46 emu/g. The catalytic efficiency of these marine-assisted MNPs was evaluated in a one-pot three-component reaction involving isatoic anhydride, aromatic aldehydes, and amines under ultrasonic conditions. Under optimal conditions, a low dose of 1.5 mg of biobased magnetite nanoparticles yielded dihydroquinazolin-4(1H)-One products with up to 95 % efficiency in a brief duration of 15 min at an ultrasonic power intensity of 130 W. Different directing groups were investigated, and control experiments were carried out to enhance the understanding of the reaction mechanism. The obtained results highlight the synergistic effect of marine grass-mediated magnetic nanocatalysts combined with ultrasonic-assisted synthesis in developing sustainable green methodologies in organic synthesis.

## Graphical abstract

**Figure:**
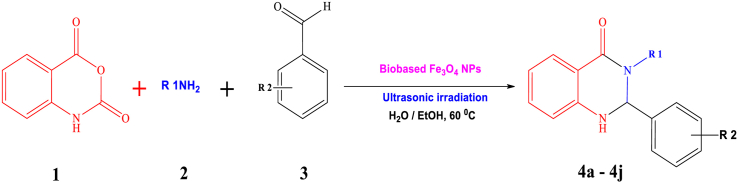

## 1 Introduction

**Scheme 1:**
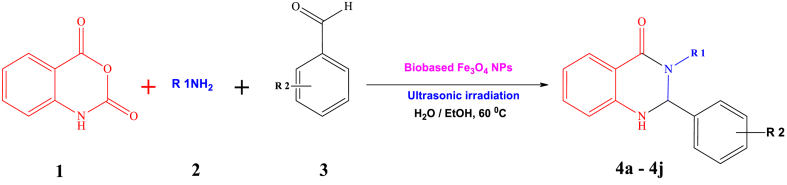
Ultrasound-assisted synthesis of 2,3-diphenyl-2,3-dihydroquinazolin-4(1H)-One derivatives 4a-j through a one-pot three-component reaction, using marine-based Fe_3_O_4_ NPs as catalysts.

Ultrasonic-assisted creating organic compounds has garnered significant attention in recent years as a powerful tool that that merges green chemistry principles with effective chemical synthesis [1]. This innovative approach utilizes high-frequency sound waves to enhance reaction rates, improve yields, and promote more sustainable chemical processes. By incorporating ultrasonic technology into organic synthesis, scientists strive to reduce environmental footprints and enhance the effectiveness of chemical reactions [2]. A significant benefit of ultrasonic-assisted synthesis lies in its capacity to hasten reaction speeds. The application of ultrasonic waves cause the creation and swift collapse of tiny bubbles, a phenomenon known as cavitation [3]. This process generates localized high temperatures and pressures, facilitating the breaking of chemical bonds and significantly speeding up reaction kinetics. As a result, reaction times can be reduced, leading to more efficient production of organic compounds. Aligned with the tenets of green chemistry, ultrasonic-assisted synthesis also enables the reduction of energy consumption [4]. The rapid and efficient reaction kinetics facilitated by ultrasonic waves often allow for lower temperatures and milder reaction conditions. This energy-saving aspect contributes to the overall sustainability of the process. Thus, ultrasonic-assisted synthesis of organic compounds signifies a notable progression in the realm of green chemistry [5]. By harnessing the power of sound waves, this technique offers improved reaction efficiency, reduced energy consumption, enhanced selectivity, and minimized use of hazardous substances [6]. Heterocyclic compounds have attracted considerable interest in the realm of medicinal chemistry due to their diverse array of biological impacts and potential as candidates for pharmaceutical applications [[7], [8], [9]]. Within this group of heterocycles, 2,3-diphenyl-2,3-dihydroquinazolin-4(1H)-one derivatives have surfaced as encouraging frameworks exhibiting a broad spectrum of pharmacological attributes, encompassing antimicrobial, anticancer, anti-inflammatory, and antiviral capabilities (Scheme 1) [9]. Their structural diversity makes them attractive targets for the synthesis of novel drug candidates [10]. However, the traditional synthetic methods for 2,3-diphenyl-2,3-dihydroquinazolin-4(1H)-one derivatives often involve the use of toxic solvents, catalysts, and high temperatures, which pose significant challenges in terms of sustainability and safety [11]. Hence, the need for green and sustainable approaches in the synthesis of 2,3-diphenyl-2,3-dihydroquinazolin-4(1H)-one derivatives is evident [[12], [13], [14]].

Environmentally friendly production of nanoparticles composed of metals and metal oxides are shown to play a crucial role as catalyst for organic transformation [[15], [16], [17], [18], [19], [20]]. Among them, eco-friendly magnetic nanocatalysts derived from plant parts extracts and marine microorganisms have emerged as promising tools for sustainable catalysis in recent years [[21], [22], [23]]. These biobased nanocatalysts offer numerous advantages, including their eco-friendly nature, abundance, and potential for renewable synthesis. The utilization of plant extracts and marine microorganisms as precursors for magnetic nanocatalyst synthesis not only provides a sustainable source but also offers unique properties and functionalities, making them highly attractive for various catalytic applications. Eco-friendly magnetic nanocatalysts have gained interest for their efficiency, reusability, and sustainability in organic synthesis [[24], [25], [26]].

**Fig. 1:**
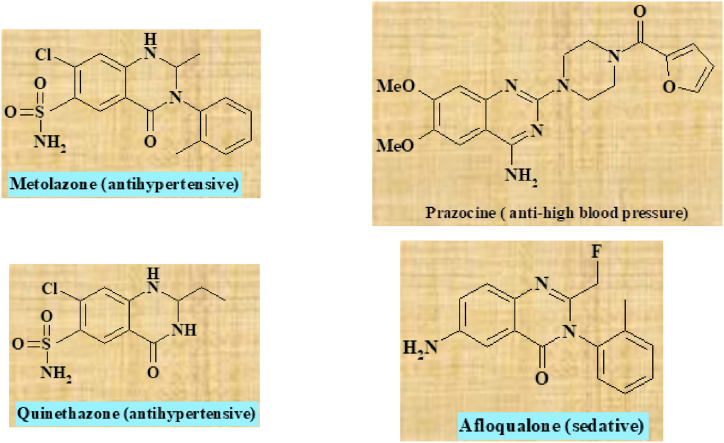
Therapeutic activities of 2,3-diphenyl-2,3-dihydroquinazolin-4(1H)-one derivatives [36].

Marine microorganisms including seaweed and seagrass have gained attention as valuable biological resources for the synthesis of nanoparticles [27,28]. Marine ecosystems are abundant in resources, and their sustainable utilization can contribute to the development of green synthesis methodologies [[28], [29], [30]]. A diverse array of nanoparticles has been synthesized utilizing eco-friendly sources, such as marine and terrestrial plant extracts, for a multitude of applications, including antimicrobial, antioxidant, and anticancer properties [[31], [32], [33]]. When plant extracts are used for nanoparticle synthesis, they serve as eco-friendly and sustainable alternatives to conventional chemical methods. These extracts contain various phytochemicals like flavonoids, phenols, alkaloids, and terpenoids, that can function as both reducing and stabilizing agents during the synthesis procedure. Marine plant extracts, derived from seaweeds and other marine vegetation, offer unique bioactive compounds that can contribute to the properties of the nanoparticles [31]. By utilizing marine biomass for production of functional nanomaterials, we can simultaneously address the issues of waste management and the synthesis of value-added products [34]. Furthermore, the integration of ultrasonic-assisted synthesis techniques with nanocatalysts has shown immense potential in enhancing reaction efficiency and reducing reaction times. Ultrasonic waves promote the dispersion of catalysts, improve mass transfer, and accelerate reaction kinetics. Consequently, ultrasonic-assisted synthesis contributes to more sustainable and efficient synthesis processes [3]. Due to a scarcity of research reports in the literature, this study represents the inaugural use of seagrass extract for the production of magnetic nanoparticles. The principal aim is to evaluate its catalytic effectiveness in organic synthesis procedures. In this investigation, we introduce a collaborative green methodology that combines a marine-sourced magnetic nanocatalyst with an ultrasonic-assisted synthesis technique to develop a fresh, sustainable, and effective synthesis process for generating 2,3-diphenyl-2,3-dihydroquinazolin-4(1H)-one derivatives (Fig. 1). We assert that the methodology employed is "green" due to the utilization of seagrass extract, a renewable and eco-friendly resource known to induce minimal environmental impact and offer sustainability benefits compared to conventional synthesis methods, abiding by green chemistry principles. This approach minimizes waste, avoids toxic reagents, is cost-effective, and operates under mild reaction conditions, contributing to a more environmentally friendly synthesis process [35].

## 2 Experimental

### 2.1 Materials and methods

The materials employed in this investigation were sourced from Sigma-Aldrich and applied without further refinement. Marine grass biomass was gathered from nearby seagrass meadows situated along coastal regions. Distilled deionized water was utilized in the preparation of all aqueous solutions. The structural configuration of the magnetic nanoparticles (MNPs) was evaluated utilizing an X-ray powder diffraction (XRD) diffractometer (Cu Ka radiation, λ = 1.5405 Å), scanning over the range of 10–80 (2θ) at a speed of 2/min. The biobased MNPs underwent UV–visible analysis with a spectrophotometer (Analytic Jena-Germany) over a wavelength range of 200–700 nm. The crystal structure of the MNPs was examined using an X-ray powder diffraction (XRD) diffractometer (Cu Ka radiation, λ = 1.5405 Å) by scanning from 10 to 80 (2θ) at a speed of 2/min. FTIR spectrum (Madison, WI, USA) was used to observe functional groups of engaged phytochemicals. Transmission electron microscopy (TEM) was employed to examine the morphology and particle size, operating at 200 kV with a Leo 912 AB instrument. The elemental composition of a sample was analysed using X-ray energy dispersive spectroscopy (EDX). Thermal behaviour of the materials was studied through thermogravimetric analysis (TGA, PerkinElmer, USA). The synthesized products' melting points were determined by employing the electrothermal IA9200 equipment to track their temperatures. Silica gel SILG/UV 254 and 365 plates were used for thin-layer chromatography (TLC) to track the reaction advancement and assess the purity of the products.

.Finally, nuclear magnetic resonance (NMR) spectroscopy was utilized to explore the composition and molecular structure of the products.

### 2.2 Extraction process of marine seagrass (*Halodule uninervis*)

To obtain a concentrated extract rich in bioactive compounds an ultrasonic-assisted solvent extraction method was utilized [37]. The gathered marine grass was washed extensively with distilled water and then gently agitated in water overnight to confirm thorough cleaning and elimination of undesired particles. Subsequently, the seagrass biomass was evenly distributed in a shaded location on a flat tray surface with adequate air flow and left to dry for a week until it became completely dry. The dried seagrass was then ground into a homogeneous powder using a mortar and pestle. Subsequently, the dried seagrass was pulverized into a consistent powder using a mortar and pestle. The dried seagrass was subsequently pulverized into a uniform powder using a mortar and pestle. For the extraction process, 5 g of the seagrass powder was blended with 200 ml of distilled water in a 250 ml beaker. The mixture underwent vigorous stirring by a magnetic stirrer at 300 rpm for a duration of 2h to extract secondary metabolites and organic components from the marine grass biomass. Afterward, the mixture was sonicated at 70 °C for 20 min. After cooling down to ambient temperature, the resulting crude extract was centrifuged for 10 min at 10000 rpm. The resultant supernatant was then used for further experiments.

### 2.3 Green synthesis of magnetite nanoparticles (Fe_3_O_4_ NPs)

Magnetic nanoparticles (Fe_3_O_4_ NPs) were synthesized by combining of 0.1 M solution of FeCl_3_.6H_2_O with an extract from marine grass in a 1:1 vol ratio (50:50 ml). The blend was agitated for a duration of 40 min under magnetic stirring at the benign temperature of 60 °C. Subsequently, it was left at room temperature for an additional 15 min. The colour changes of the iron and extract mixture from yellow to black were observed [38]. The colloidal suspensions obtained were then subjected to centrifugation and multiple washes with a 50 % water-ethanol mixture. Finally, the magnetic nanoparticles were subjected to drying in an oven set at 80 °C for 2 h to achieve the desired product.

General procedure for magnetite NPs-catalyzed synthesis of 2,3-diphenyl-2,3-dihydroquinazolin-4(1H)-One derivatives.

A solution containing isatoic anhydride (**1**,1 mmol), amine (**2**,1 mmol), and aromatic aldehyde (**3**, 1 mmol) was prepared in a 25 ml Erlenmeyer flask. Heterogeneous iron oxide NPs (0.005 mmol, 1.5 mg) were added to the solution in 10 ml of water. The mixture was then subjected to ultrasound irradiation (US) at a temperature of 60 °C for 15 min, as shown in Scheme 1. The completion of the reaction was monitored using TLC. Once the reaction was finished, the mixture was cooled to room temperature, and 50 ml of water was added to induce precipitation of the desired product. The product underwent filtration and drying. Following this, the precipitate was rinsed with 20 ml of pure ethanol. In order to assess the Fe_3_O_4_ nanocatalyst's ability to be reused, it was extracted from the solution through an external magnet and dried overnight at 80 °C. In order to acquire pure substances, the sediment was heated in 15 ml of ethanol for 5 min. The resultant crystalline materials were scrutinized using spectroscopic methods like FTIR, elemental analysis, and ^1^H and ^13^C NMR to ascertain their characteristics and composition (Figs. S1–3 in the supplementary materials).

## 3 Result and discussion

### 3.1 UV–vis analysis

**Fig. 2:**
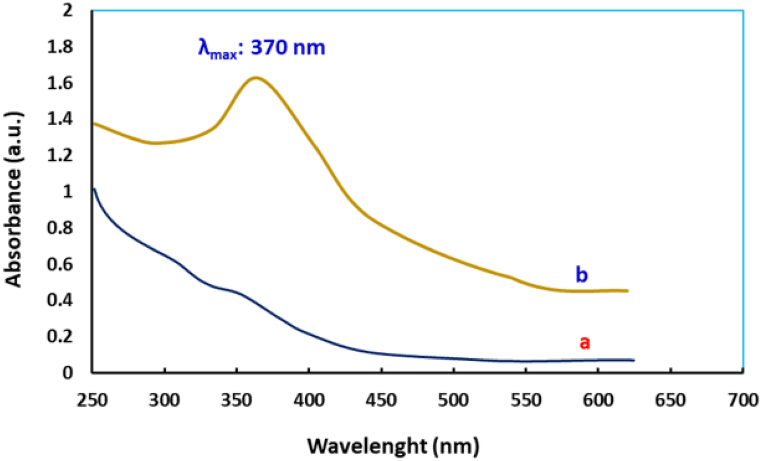
UV–vis spectra of (a) Seagrass Extract and (b) biosynthsized Fe3O4 Nps within seagrass extract.

Fig. 2 illustrates the UV–vis spectrum of the newly prepared MNPs and the aqueous extract from marine grass. In contrast to pristine grass extract, the advent absorption peak at wavelength of 370 nm which may be assigned to the surface plasmon absorption of Fe_3_O_4_ NPs [39]. This peak corresponds to the absorption of light by the magnetite nanoparticles, indicating their presence and confirming their formation. This result aligns with our earlier research, where we found an absorption peak at 375 nm using potato extract [40]. It is worth noting that the absorption peak of Fe_3_O_4_ nanoparticles can indeed vary depending on the synthesis methodology, particle size, particle shape, and surface modifications [41].

### 3.2 XRD pattern

**Fig. 3:**
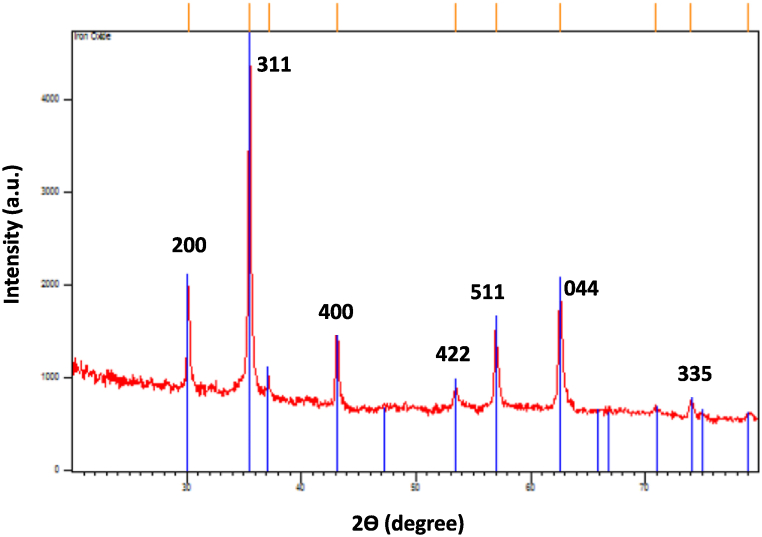
XRD pattern of biosynthesized magnetite NPs using marine grass extract.

X-ray powder diffraction was employed to investigate the phase identification and crystalline structures of the nanoparticles. The XRD patterns depicted in Fig. 3 are from the MNPs synthesized using marine grass extract. These patterns exhibit clear peaks at 2θ values of 30.4°, 35.56°, 43.5°, 54.1°, and 57.4°, which align with the crystal planes of (200), (311), (511), and (440) of crystalline iron oxide powder, in accordance with the specified JCPDS (Joint Committee on Powder Diffraction Standards) No. 980111228. It is a reference code that identifies the specific crystalline structure of Fe_3_O_4_. These results indicate that the NPs possess an inverse spinel crystal structure with a cubic close-packed phase of magnetite, which aligns with the X-ray diffraction standard for magnetite nanoparticles [38,42]. The mean particle dimension of magnetite nanoparticles at the most pronounced peak (2θ) was calculated to be 18 nm employing the Debye-Scherrer equation (1):

(1) $$d=\frac{K\lambda }{\beta .\cos\;Ɵ}$$

Here, d signifies the crystal's particle size, k stands for the Sherrer constant (0.9), λ denotes the X-ray wavelength (0.15406 nm), β represents the XRD peak width at half-height, and θ corresponds to the Bragg diffraction angle.

### 3.3 FTIR analysis

**Fig. 4:**
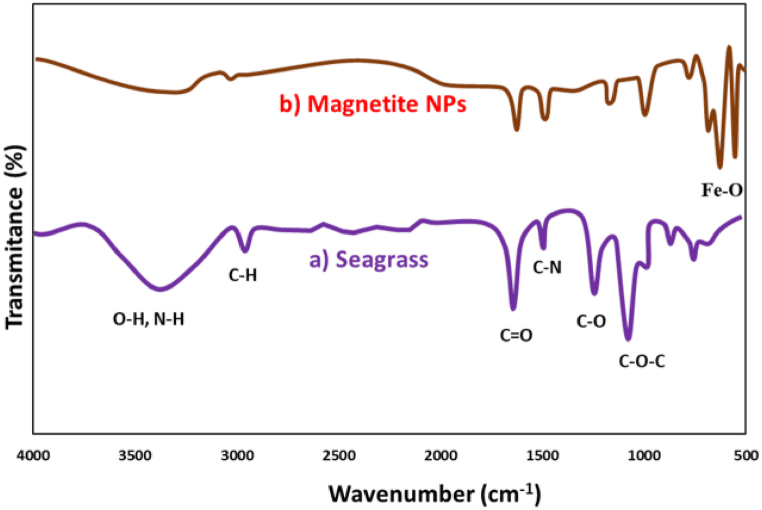
(a) FTIR spectra of seagrass extract (*Halodule uninervis*) and (b) seagrass-mediated synthesized magnetite NPs.

The active components' functional groups were determined through FTIR spectroscopy, which examined the peak values within the infrared radiation spectrum. Fig. 4 presents the FTIR spectra of seagrass powder (Fig. 4a) and seagrass-biopepared Fe_3_O_4_ NPs (Fig. 4b). Seagrass tissues harbor a diverse range of secondary metabolites, including lipids, phenylpropanoid derivatives, protein, phenol, fiber, flavonoid, and tannin [43,44]. Consequently, the FTIR analysis revealed distinct vibrational stretching peaks in the seagrass extract at specific wavenumbers: 3304 cm^−1^ (-NH and -OH), 1630 cm^−1^ (carbonyl groups of proteins or amides), 2931 cm^−1^ (C-H aliphatic groups), and 1352 cm^−1^ (C-N single bond). Additionally, the vibrational bands observed at 1190 cm^−1^ and 1080 cm^−1^ corresponded to alcoholic C-O and C-O-C groups of tannins, respectively. Regarding the biofabricated Fe_3_O_4_ NPs, significant changes in the position and intensities of absorption peaks were observed. Notably, sharp absorption bands at 520 cm^−1^ and 599 cm^−1^ confirm the presence of the Fe–O bond in magnetite, indicating the formation of magnetic Fe_3_O_4_ NPs [45]. Presumably, the functional groups present in electron-rich phytochemicals derived from seagrass show promise in their capacity to serve dual roles as reducing agents and capping agents. This capability facilitates the transformation of Fe ions into MNPs and is crucial for their stabilization within the seagrass extract [[46], [47], [48]].

### 3.4 TEM study

**Fig. 5:**
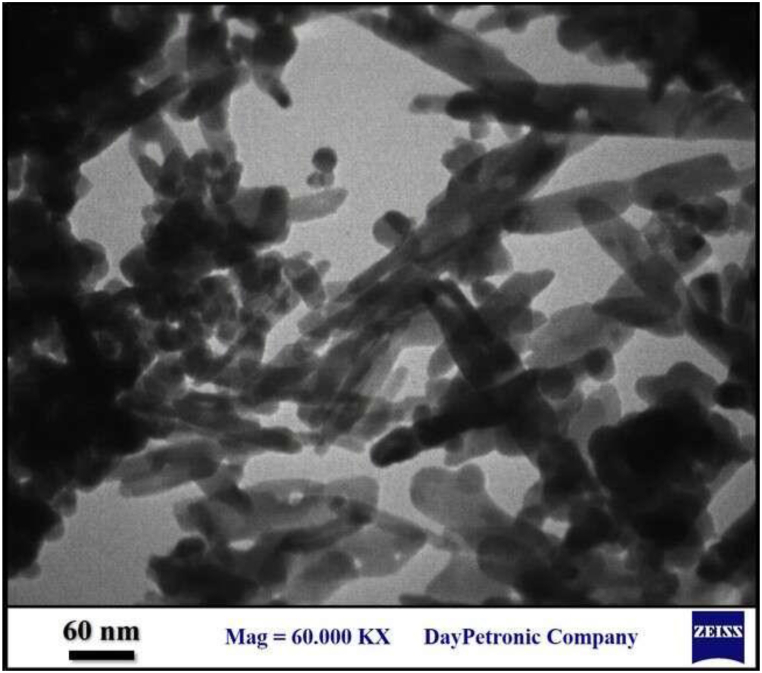
TEM image of biosynthesized magnetite NPs using marine grass extract at magnifications of 60.000 KX.

A TEM study was conducted to gain insight into the size and morphological characteristics of seagrass-mediated magnetite NPs. The biobased magnetite NPs exhibited predominantly rod-shaped structures, having an average dimension of 21 nm as depicted in Fig. 5. This finding aligns with previous literature studies that have also reported the rod morphology of Fe_3_O_4_ NPs using different methods [49,50]. For instance, prasad et al. prepared iron oxide magnetic NPs with nanorod structure using an aqueous extract of Pomegranate leaves [51].

### 3.5 Magnetic properties of biofabricated iron oxide NPs

**Fig. 6:**
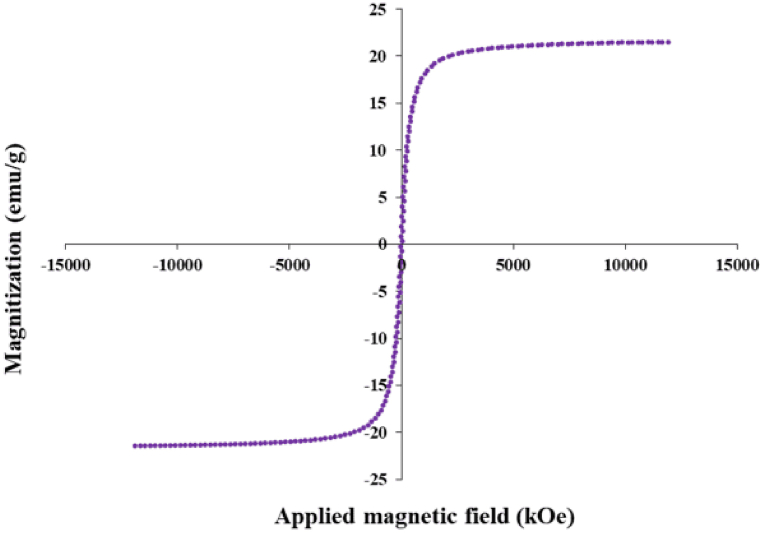
Magnetization curve of green Fe_3_O_4_ NPs.

Exploring the magnetic properties of Fe_3_O_4_ NPs involved performing magnetization assessments utilizing a Vibrating Sample Magnetometer (VSM). The saturation magnetization (Ms) of the Fe_3_O_4_ NPs was ascertained to be 21.46 emu/g while the values of remnant magnetization (Mr) and Hc (coercive force) were 4.18 emu/g and 22 Oe, respectively. The absence of a hysteresis loop in the magnetization curve indicates that the iron oxide nanoparticles exhibited superparamagnetism (Fig. 6). It is widely recognized that magnetic materials display superparamagnetic behaviour when their size is below a critical threshold, typically around 35 nm [52]. In a study by Mahdavi et al., the saturation magnetization value of Fe_3_O_4_ NPs synthesized using brown seaweed extract was recorded at 22.1 emu/g which closely aligns with our findings. It is important to highlight that the magnetic characteristics of NPs may differ based on their morphology, size, and preparation method used, leading to different saturation magnetization (Ms) values reported in the literature [51,53].

### 3.6 TGA analysis

**Fig. 7:**
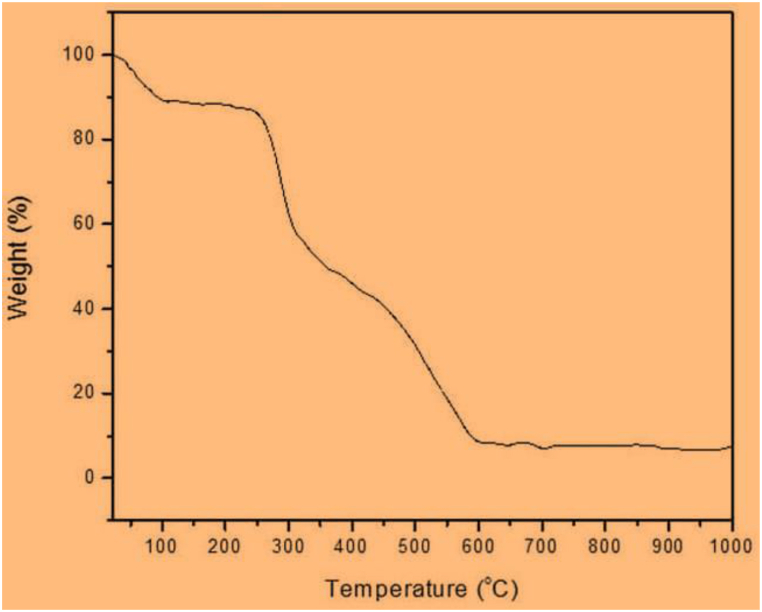
TGA curve of biosynthesized magnetic nanoparticles.

The result of TGA curve depicted in Fig. 7 reveals there are three weight loss steps. The initial stage, occurring between 10 and 125 °C, is linked to the presence of water molecules adsorbed on the nanoparticles' surface. Subsequently, the second stage, falling within the 250–350 °C range, is associated with the preliminary breakdown of phytochemical compounds. The decomposition of the carbon-carbon skeleton of organic materials is observed in the range 400–600 °C. Over this temperature, the changes in sample structure approximately remain constant showing the thermal stability of the produced MNPs.

### 3.7 Catalytic activity of green Fe_3_O_4_ NPs

**Scheme 2:**
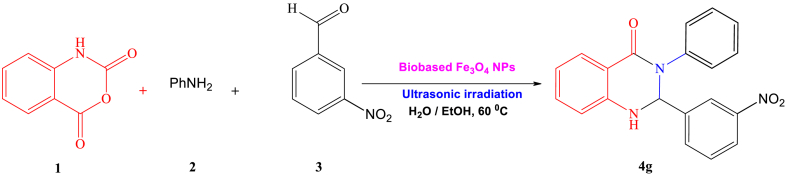
synthetic rout of product 4g using optimal amount of seagrass-derived magnetite nanocatalyst.

In our pursuit of environmentally friendly methods to synthesize 2,3-diphenyl-2,3-dihydroquinazolin-4(1H)-One derivatives under optimal conditions, we explored different quantities of Fe_3_O_4_ catalyst through a straightforward three-component reaction process. This method entailed the use of isatoic anhydride (**1**,1 mmol), amine (**2**,1 mmol), and aromatic aldehyde (**3**, 1 mmol) as representative reactants, coupled with ultrasonic exposure and water as a solvent (Scheme 2). The blend was warmed to 60 °C for the required duration. The advancement of the reaction was tracked using TLC (ethyl acetate/n-hexane, 1:5). Upon completion of the reaction, hot ethanol (15 ml) was introduced, followed by filtration of the mixture. The purified 2-(3-nitrophenyl)-3-phenyl-2,3-dihydroquinazolin-4(1H)-one (**4g**) was acquired through recrystallization from ethanol. As indicated in Table 1, Table 2, the maximum product yield (95 %) was achieved at a molar concentration of nanoparticles of 0.005 mmol (1.15 mg). However, increasing the quantity of magnetite nanocatalyst did not lead to notable enhancements in either the yield or the reaction pace (see Table 2).

**Table 1:** Enhancing the efficiency of a catalytic dose of biobased Fe_3_O_4_ NPs in the aqueous production of **4g**.

Entry	Catalyst dose (mmol)	Time (min)	Yield (%)
1	0.00	15	minimal
2	0.001	15	75
3	0.003	15	88
4	0.005	15	95
5	0.009	15	93
6	0.01	15	91

**Table 2:** depicts the impact of different ultrasonic intensities on the synthesis of 4g.

Entry	Power intensity (W)	Time (min)	Yield (%)
1	neat	240	45
2	30	30	77
3	80	25	86
4	130	15	95
5	180	15	91
6	230	15	88

### 3.8 The impact of ultrasonic power intensity on chemical reactions

The influence of ultrasonic power intensity on a reaction was studied, employing various levels of ultrasonic bath power ranging from 30 to 230 W. Surprisingly, the most significant product output of 95 % was achieved when employing an ultrasonic power intensity of 130 W in conjunction with a short reaction duration of 15 min. Conversely, utilizing lower power intensities of 30 W and 80 W resulted in diminished product yields of 77 % and 86 % respectively, coupled with extended reaction durations of 30 and 25 min. This phenomenon can be explained by the diminished acoustic cavitation produced by the sound waves, leading to a less effective creation of reactive species and slower rates of reaction. Conversely, when operating at higher power intensities of 180 W and 230 W, although the reaction times remained constant at 15 min (as with 130 W), the product yields declined to 91 % and 88 % respectively. The decrease in product yield can be linked to the overpowering intensity of cavitation, which may result in the creation of undesired byproducts or the breakdown of reactants, thus impeding the intended reaction. Interestingly, without the use of ultrasonic irradiation, the reaction proceeded less efficiently overall. This underscores the crucial function of ultrasonic waves in facilitating the generation of reactive species and accelerating the reaction pace. Therefore, the optimal ultrasonic power intensity for maximizing the efficiency of the reaction was found to be 130 W. At this level of intensity, the acoustic cavitation exhibited ample strength to aid in the creation of reactive species and hasten the reaction speed, culminating in a notable product yield achieved in a brief reaction period (see Tabfig.le 2).

### 3.9 Efficiency evaluation of magnetite nanocatalyst

In order to assess how effectively Fe_3_O_4_ NPs perform as catalysts, a series of experiments were conducted in a water-based solution using different catalysts. The effectiveness of these catalysts was assessed by evaluating the turnover frequency (TOF) and turnover number (TON). TOF quantifies the number of catalytic reactions that can be executed by a single active site of the catalyst, whereas TON signifies the quantity of catalytic reactions achievable by a defined quantity of catalyst before its activity diminishes within a specific timeframe. These factors serve as crucial benchmarks for assessing a catalyst's efficacy in a specific reaction. The derived TOF and TON data indicated that sea grass extract-mediated MNPs exhibited enhanced stability and heightened efficiency when juxtaposed with alternative catalysts like chemically produced bare magnetite nanoparticles **(see** Table 3**)**. However, the magnetite catalysts from both commercial and biogenic sources (entries 4 and 5) yielded higher results when compared to traditional acid catalysts (entries 1–3). It is evident that the surface of biogenic MNPs produced by marine grass extract may feature unique functional groups or active sites that facilitate a more robust interaction with reactant molecules. This enriched surface chemistry enhances the catalyst's efficiency, resulting in increased yields and accelerated reaction rates. Thus, the integration of organic components from seagrass on the catalyst's surface contributes significantly to enhancing the catalytic capabilities of MNPs. It is crucial to underline that in the absence of a catalyst, merely a trivial quantity of the targeted products was acquired even under ideal conditions (entry 6). This finding aligns with our prior research, which highlighted the superior catalytic performance of biogenic tin oxide [3], gold [54], and nickel oxide [28] nanocatalysts showcased enhanced catalytic efficacy in contrast to their conventional peers.

**Table 3:** Comparative analysis of various catalysts for preparation of **4g** product.

Entry	Catalyst	Time (min)	TON	TOF	Yield (%)
**1**	AlCl_3_	120	6400	53.33	32
**2**	bulk iron oxide	140	9000	64.28	45
**3**	benzoic Acid	110	12000	109.09	60
**4**	biological iron NPs	15	19000	1266.66	95
**5**	chemical iron NPs	70	16400	218.66	82
**6**	neat	overnight	–	–	trace

### 3.10 Investigating the influence of solvent type and temperature in ultrasonic treatment

To examine how temperature affects the synthesis process, we carried out the model reaction at different temperature gradients. The resultant product exhibited diverse yields, fluctuating between 87 % and 95 %, particularly when water was employed as the solvent (entries 8–10, Table 4). The results indicate that solvents with higher dielectric constants tend to yield products at lower temperatures with higher yields (entries 1–6). Nonetheless, concerning water, characterized by the highest dielectric constant (*ε* = 78.5), elevating the temperature from 40 to 60 °C had a beneficial impact on both yield and reaction duration. Subsequently, with a further rise in temperature from 60 to 90 °C, the yield decreased to 89 % (entry 11).

**Table 4:** the impact of solvent and temperature on the synthesis of the **4g** product under ultrasonic irradiation.

Entry	Solvent	Temperature (°C)	Time (min)	Yield (%)
1	(CH₃)₂SO	45	25	76
2	C_2_H_5_OH	50	30	65
3	CH_3_CN	55	45	55
4	CH_3_CO_2_C_2_H_5_	60	75	38
5	CHCl_3_	60	85	35
6	CH_3_COCH_3_	60	72	30
7	neat	60	85	45
8	H_2_O	40	25	87
9	H_2_O	50	15	92
10	H_2_O	60	15	95
11	H_2_O	80	15	89

This decrease might be ascribed to the adverse effects of elevated reaction temperatures on cavity development and longevity, causing a notable rise in vapor pressure within the liquid. As a result, the cavitation bubbles contained a higher vapor content, potentially leading to the reduction in yield. Our findings conclude that water stands out as the ideal solvent for the one-step synthesis of 2,3-diphenyl-2,3-dihydroquinazolin-4(1H)-One derivatives under ultrasonic irradiation settings. The results strongly suggest that the temperature of the water solvent plays a crucial role in achieving high yields and efficient reaction times.

### 3.11 Reusability of magnetite nanocatalyst

**Fig. 8:**
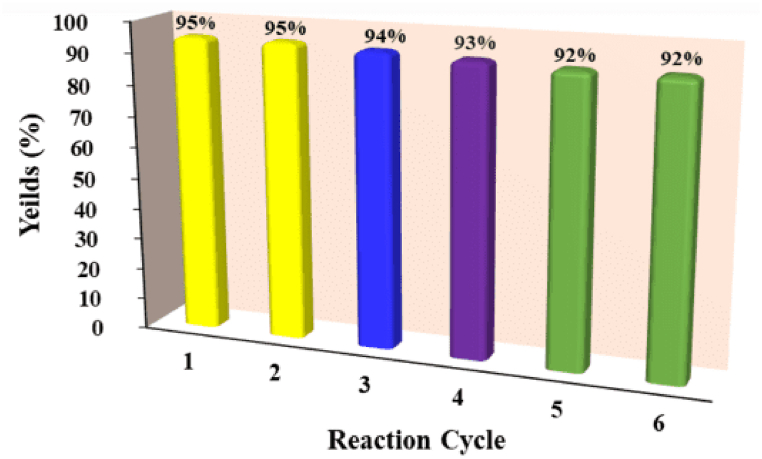
The reusability test conducted on the biogenic magnetite nanocatalyst over six conductive experiment cycles.

In order to assess the recyclability of the biobased magnetite catalyst, the nanoparticles were isolated from the reaction mixture using a magnetic stirrer. Subsequently, they underwent a comprehensive washing process with ethanol and water to guarantee a pristine catalyst devoid of any impurities. As evidence in Fig. 8 the catalytic performance of magnetite NPs remained largely unaffected even after undergoing subsequent five catalytic runs (95 %, 95 %, 94 %, 93 %, 92 %, 92 %). The initial amount of catalyst was 1.5 mg, and after 5 cycles, the final amount only decreased to 0.978 mg. This proposes that the quantity of the catalyst remained fairly stable across the recovery cycles (Fig. 8).

A comprehensive comparison was conducted to assess the efficacy of the current approach relative to diverse heterogeneous catalysts documented in literature for synthesis of 2,3-dihydroquinazolin-4(1H)-ones. The main goal was to assess the efficiency of the eco-friendly nano catalyst in terms of both output and reaction duration. The results presented in Table 5, unequivocally demonstrate that the seagrass-derive Fe_3_O_4_ nanocatalyst reveals satisfied characteristic in terms of cost, greener methodology, yield and reaction time. This compelling evidence underscores the remarkable effectiveness of the biogenic magnetite NPs as a catalytic material.

**Table 5:** a comparative analysis of the catalytic activity between seagrass-mediated Magnetite NPs and other reported catalysts.

Entry	catalyst	time	Reaction condition	yield (%)	reference
**1**	Hydroxyapatite NPs	60 min	H_2_O, 110 °C	95	[55]
**2**	KCC-1/Pr-SO_3_H	120 min	EtOH, reflux	95	[56]
**3**	Nano-In_2_O_3_	240 min	EtOH/H_2_O, 80 °C	87	[57]
**4**	Nano-NiAl_2_O_4_	15 min	MW, 45 °C	96	[11]
**5**	CoAl_2_O_4_ spinel nanocrystal	15 min	US, EtOH, 45 °C	96	[58]
**6**	Nano-ZnO	180 min	solvent-free, 70 °C	88	[59]
**7**	Biobased magnetite NPs	15 min	US, H_2_O, 60 °C	95	This work

#### 3.11.1 Evaluation of the catalytic scope of magnetite NPs

To evaluate the general efficiency of the catalyst, magnetic NPs were experimented with various functionalized organic aldehydes under diverse chemical reactfiion settings (Table 6). Despite the diverse functional groups present on the aromatic aldehydes, the target 2,3-diphenyl-2,3-dihydroquinazolin-4(1H)-One derivatives were successfully synthesized with high yields and exceptional purity. This outcome underscores the substantial impact of environmentally sustainable MNPs in expediting the reaction. Moreover, as illustrated in Table 6, the incorporation of the NO_2_ group at the meta-position of the aryl aldehyde exhibited a noteworthy positive influence on the overall reaction performance, leading to an impressive yield of 96 % (entry 7).

**Table 6:**
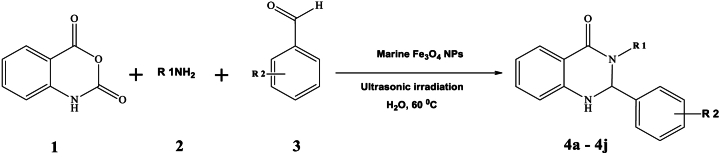
the synthesis of 2,3-diphenyl-2,3-dihydroquinazolin-4(1H)-One derivatives using ultrasonic-mediated biobased magnetite nanocatalyst.

Entry^a^	R _1_	R _2_	Product		Yield^b^ (%)	Melting point (°C, lit.)
1	Ph	H		**4a**	94	218-220 (220–222) [60]
2	Ph	4-OMe		**4b**	90	205-207 (204–206) [60]
3	Ph	4-Cl		**4c**	90	210-212 (228–230) [60]
4	4*-* OMePh	H		**4d**	88	217-219 (215–216) [61]
5	4*-* OMePh	4*-*Me		**4e**	85	154-156 (151–153)^44^
6	NHPh	2-Cl		**4f**	92	180–182
7	NHPh	3-NO_2_		**4g**	95	179-181 (178–180) [62]
8	NHPh	3-Br		**4h**	88	165–167
9	NHPh	4-OH		**4i**	88	164-166 (166) [63]
10	NHPh	3-Br,2- OH		**4j**	90	179–181

a Reaction conditions: Isatoic anhydride (1 mmol), amino (1 mmol), aromatic aldehyde (1 mmol), and Fe_3_O_4_ NPs (0.005 mmol).

b Isolated yield.

**Scheme 3:**
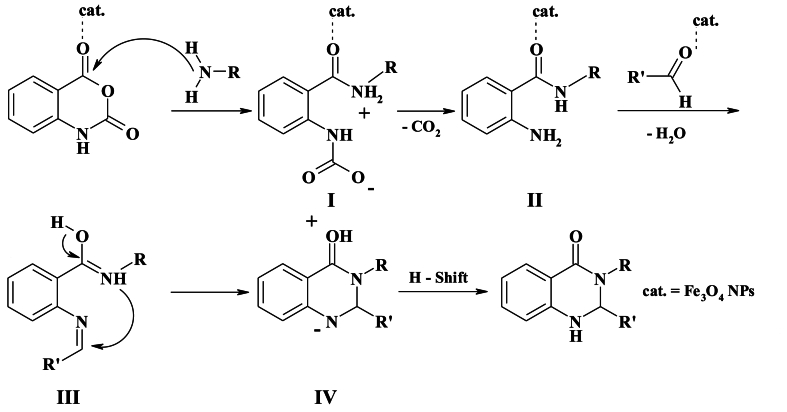
a plausible mechanism for the synthesis of 2,3-dihydroquinazolin-4(1H)-ones.

Initially, the isatoic anhydride undergoes activation by magnetite NPs. This activation is followed by the attack of an N-nucleophilic amine on the carbonyl group, leading to the formation of intermediate I. Subsequently, decarboxylation takes place, resulting in the generation of 2-amino-N-substituted-benzamide (II). In this process, magnetic Fe_3_O_4_ NPs act as Lewis's acid, playing a significant role in enhancing the electrophilic nature of the aldehydes. Following this, the activated aldehyde reacts with intermediate II, giving rise to intermediate III. Finally, through an intramolecular cyclization, intermediate III is converted into product IV (Scheme 3).

## 4 Conclusion

We have successfully developed a highly efficient one-pot method for synthesizing a diverse range of 2,3-diphenyl-2,3-dihydroquinazolin-4(1H)-One derivatives. This synthesis involves a three-component condensation reaction conducted in the presence of ultrasonic irradiation and a green catalyst consisting of seagrass extract-mediated magnetic Fe_3_O_4_ NPs, all performed in water. The greater values of TON and TOF confirmed the higher stability and catalytic efficiency of heterogenic biobased magnetic nanocatalysts compared to the studied counterparts in this study. The combination of ultrasound power and a low dose of magnetite catalyst (1.5 mg) exhibited a synergistic effect, resulting in an accelerated reaction rate (15 min) and facilitating the desired transformation of organic compounds (85–95 %) under mild condition (60 °C). Owing to readily recovered by magnetic separation and proper reusability (six runs), the developed procedure contributes to the development of environmentally friendly processes and aligns with the growing demand for sustainable and efficient methodologies in chemical synthesis.

## Data availability

All data generated or analysed during this study are included in this article.

## CRediT authorship contribution statement

**Foad Buazar:** Writing – review & editing, Writing – original draft, Validation, Software, Methodology, Conceptualization. **Mohammad Hosein Sayahi:** Validation, Supervision, Methodology, Investigation, Formal analysis.

## Declaration of competing interest

The authors declare that they have no known competing financial interests or personal relationships that could have appeared to influence the work reported in this paper.
