# Supplementary material for: Marine-derived magnetic nanocatalyst in sustainable ultrasound-assisted synthesis of 2,3-diphenyl-2,3-Dihydroquinazolin-4(1H)-One derivatives
Source: Heliyon. 2024 Oct 5;10(19):e38948. doi: 10.1016/j.heliyon.2024.e38948 (PMC11492587; doi:10.1016/j.heliyon.2024.e38948)
Supplement: Multimedia component 1 [file mmc1.docx]

**Brand-New Marine-Derived Magnetic Nanocatalyst in Sustainable Ultrasound-Assisted Synthesis of 2,3-Diphenyl-2,3-Dihydroquinazolin-4(1H)-One Derivatives**

**Foad Buazar*^a^ and Mohammad Hosein Sayahi*^b^**

^a^Department of Marine Chemistry, Khorramshahr University of Marine Science and Technology, P.O. Box 669, Khorramshahr, Iran

^b^Department of Chemistry, Payame Noor University, Tehran, Iran

*****Corresponding author: [fb@kmsu.ac.ir](mailto:fb@kmsu.ac.ir) (F. Buazar)

Tel.: (0098) 9161150684; Fax: (0098) 6153533322


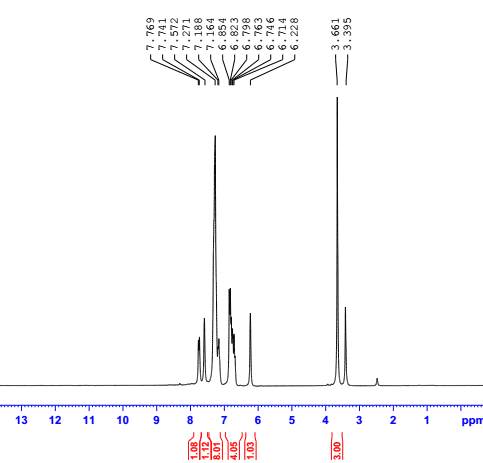


**a**


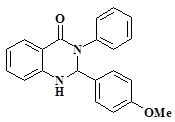


**4b**


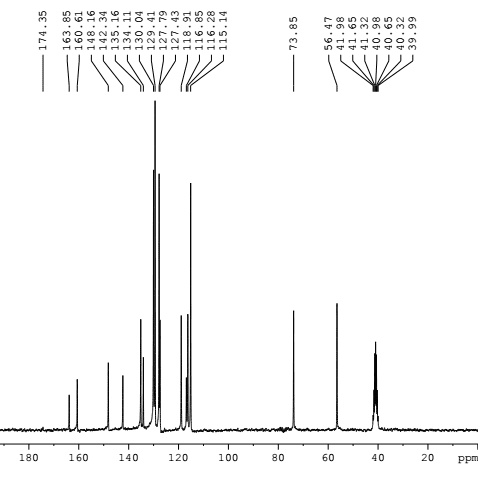


**b**


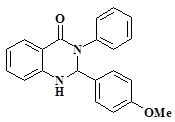


**4b**

**2-(4-Methoxyphenyl)-3-phenyl-2,3-dihydroquinazolin-4(1H)-one** (**4b**): White solid; mp 205–207 °C; FT-IR (KBr): ν_max_/cm^−1^ 3447, 3297, 1633, 1611, 1508, 1389, 1250, 1171, 835, 694, 605; ^1^H NMR (250 MHz, DMSO-d_6_, ppm) δ 3.66 (s, 3H, OMe), 6.23 (s, 1H, CH), 6.71–6.85 (m, 4H, Ar), 7.17–7.28 (m, 8H, Ar), 7.57 (s, 1H, Ar), 7.75 (d, 1H, NH); ^13^C NMR (63 MHz, DMSO-d_6_, ppm) δ 56.4, 73.8, 115.1, 116.3, 116.9, 118.9, 127.4, 127.8, 129.4, 130.0, 134.1, 135.1, 142.3, 148.1, 160.6, 163.8; 174.3; *Anal*. calcd. For C_21_H_18_N_2_O_2_: C, 76.34; H, 5.49; N, 8.48. Found: C, 76.40; H, 5.55; N, 8.55.

**Fig. S1.** (a)^1^HNMR and (b) ^13^CNMR spectra and other relevant analyses of **4b** product.


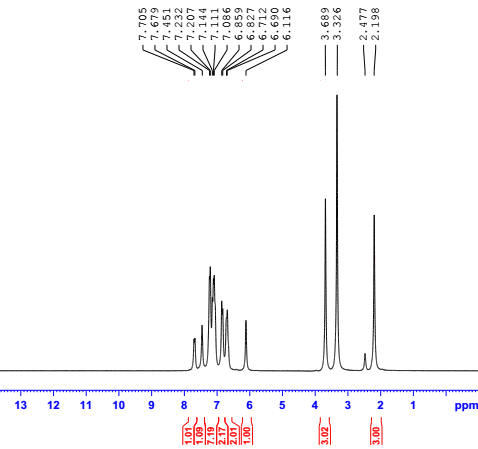


**a**


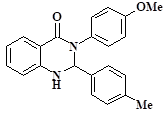


**4e**


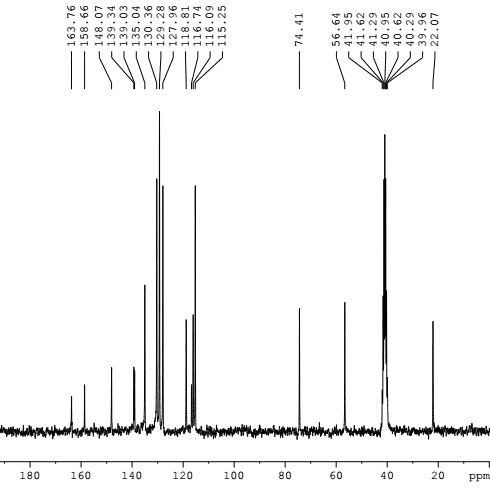


**b**


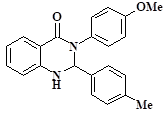


**4e**

**3-(4-Methoxyphenyl)-2,3-dihydro-2-p-tolylquinazolin- 4(1H)-one** (**4e**). White powder; mp 154- 156 °C; FT-IR (KBr): ν_max_/cm^−1^ 3291, 1633, 1611, 1510, 1486, 1394, 1244, 1026, 834, 760, 699; ^1^H NMR (250 MHz, DMSO-d_6_, ppm) δ 2.20 (s, 3H, Me), 3.69 (s, 3H, OMe), 6.12 (s, 1H, CH), 6.69-6.86 (m, 4H, Ar), 7.09-7.23 (m, 9H, Ar), 7.45 (s, 1H, Ar), 7.69 (d, 1H, NH); ^13^C NMR (63 MHz, DMSO-d_6_, ppm) δ 22.0, 56.6, 74.4, 115.2, 116.0, 116.7, 118.8, 127.9, 129.3, 130.4, 135.0, 139.0, 139.3, 148.0, 158.7, 163.8; *Anal*. calcd. For C_22_H_20_N_2_O_2_: C, 76.72; H, 5.85; N, 8.13. Found: C, 76.65; H, 5.90; N, 8.18.

**Fig. S2.** (a)^1^HNMR and (b) ^13^CNMR spectra and other relevant analyses of **4e** product.


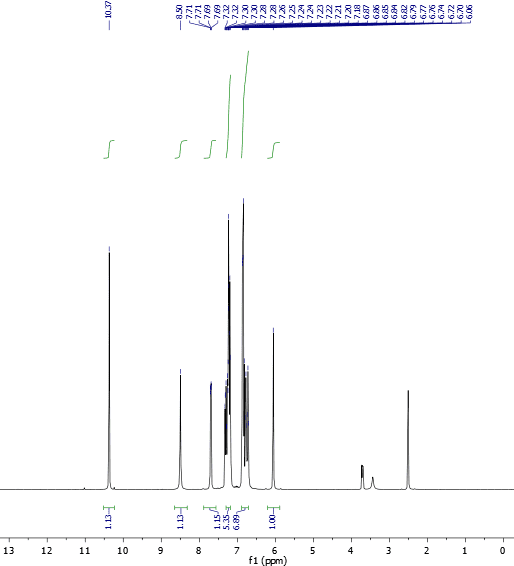


**a**


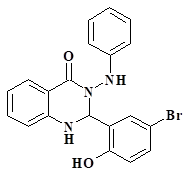


**4j**


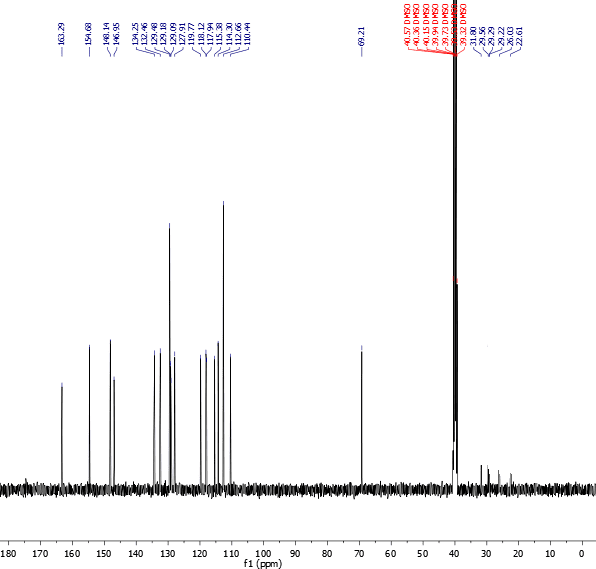


**b**


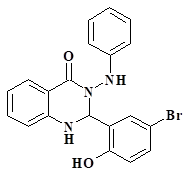


**4j**

**2-(5-bromo-2-hydroxyphenyl)-3-(phenylamino)-2,3-dihydroquinazolin-4(1*H*)-one (4j)**

White solid; mp 179–181 °C; FT-IR (KBr): ν_max_/cm^−1^ 3447, 3287, 1633, 1611, 1508, 1486, 1389, 1250, 1029, 835, 694; ^1^H NMR (400 MHz, DMSO-*d*_6_) δ 6.06 (d, *J* = 2.4 Hz, 1H), 6.52–7.02 (m, 6H, Ar), 7.13–7.40 (m, 6H, Ar), 7.55–7.84 (m, 1H, NH), 8.51 (s, 1H, NH), 10.37 (s, 1H, OH);^13^C NMR (101 MHz, DMSO-d_6_, ppm) δ 163.3, 154.7, 148.1, 146.9, 134.3, 132.5, 129.5, 129.2, 129.0, 127.9, 119.8, 118.1, 117.9, 115.4, 114.3, 112.7, 110.4, 69.2; *Anal*. calcd. For C_20_H_16_BrN_3_O_2_: C, 58.55; H, 3.93; N, 10.24. Found: C, 58.32; H, 4.15; N, 10.37.

**Fig. S3.** (a)^1^HNMR and (b) ^13^CNMR spectra and other relevant analyses of **4j** product
